# Phase III randomised trial of doxorubicin-based chemotherapy compared with platinum-based chemotherapy in small-cell lung cancer

**DOI:** 10.1038/sj.bjc.6604480

**Published:** 2008-07-29

**Authors:** S Baka, R Califano, R Ferraldeschi, L Aschroft, N Thatcher, P Taylor, C Faivre-Finn, F Blackhall, P Lorigan

**Affiliations:** 1 Department of Medical Oncology, Christie Hospital NHS Foundation Trust, Wilmslow Road, Manchester M20 4BX, UK; 2 Pulmonary Oncology Unit, University Hospital of South Manchester NHS Foundation Trust, South Moors Road, Manchester M23 9LT, UK; 3 Department of Clinical Oncology, Christie Hospital NHS Foundation Trust, Wilmslow Road, Manchester M23 4BX, UK

**Keywords:** small-cell lung cancer, chemotherapy, randomised clinical trial, cisplatin, doxorubicin

## Abstract

This randomised trial compared platinum-based to anthracycline-based chemotherapy in patients with small-cell lung cancer (limited or extensive stage) and ⩽2 adverse prognostic factors. Patients were randomised to receive six cycles of either ACE (doxorubicin 50 mg/m^2^ i.v., cyclophosphamide 1 g/m^2^ i.v. and etoposide 120 mg/m^2^ i.v. on day 1, then etoposide 240 mg/m^2^ orally for 2 days) or PE (cisplatin 80 mg/m^2^ and etoposide 120 mg/m^2^ i.v. on day 1, then etoposide 240 mg/m^2^ orally for 2 days) given for every 3 weeks. For patients where cisplatin was not suitable, carboplatin (AUC6) was substituted. A total of 280 patients were included (139 ACE, 141 PE). The response rates were 72% for ACE and 77% for PE. One-year survival rates were 34 and 38% (*P*=0.497), respectively and 2-year survival was the same (12%) for both arms. For LD patients, the median survival was 10.9 months for ACE and 12.6 months for PE (*P*=0.51); for ED patients median survival was 8.3 months and 7.5 months, respectively. More grades 3 and 4 neutropenia (90 *vs* 57%, *P*<0.005) and grades 3 and 4 infections (73 *vs* 29%, *P*<0.005) occurred with ACE, resulting in more days of hospitalisation and greater i.v. antibiotic use. ACE was associated with a higher risk of neutropenic sepsis than PE and with a trend towards worse outcome in patients with LD, and should not be studied further in this group of patients.

The last decades have seen considerable efforts to improve the outcome for patients with small-cell lung cancer (SCLC) but progress has been slow (Govindan *et al*, 2006). Small-cell lung cancer is highly sensitive to chemotherapy and combination regimens have been the cornerstone of treatment since the 1970s. Characterisation of SCLC into limited (LD) and extensive disease (ED) as proposed originally by the Veterans Administrator Lung Cancer Group and revised by the International Association for the Study of Lung Cancer (IASCL) has been the basis of treatment choice for a number of years (Mountain, 1986; Zelen, 1973). A number of other independent prognostic factors including performance status (PS) and biochemical parameters (eg serum sodium, alkaline phosphatase and serum lactate dehydrogenase) have been identified and prognostic scores using these variables can reliably identify patients with good, intermediate or poor outcome (Buccheri and Ferrigno, 2004; Cerny *et al*, 1987; Sagman *et al*, 1991; Thatcher *et al*, 1995).

When the current trial was designed in 1999, trials comparing platinum-based with anthracycline-based chemotherapy in SCLC were ongoing and the importance of concurrent chemoradiotherapy was just beginning to be understood. The ACE combination was still widely used in Europe (Thatcher *et al*, 2000; Sambrook and Girling, 2001) and was a reference regimen for the European Organisation for Research and Treatment of Cancer (EORTC) Lung Group (Ardizzoni *et al*, 2002; Giaccone *et al*, 1993; Postmus *et al*, 1996). Median survivals of 9–11 months with 1-year survival of 30–40% were reported in trials of both LD and ED patients with good PS (Giaccone *et al*, 1993; Thatcher *et al*, 2000; Urban *et al*, 1999). However, treatment with ACE was associated with significant neutropenia that contributed to infection-related morbidity and mortality (Bunn *et al*, 1986; Postmus *et al*, 1996; Thatcher *et al*, 2000). Cisplatin and etoposide (PE) were widely used in North America, with similar survival rates to those reported for cyclophosphamide, doxorubicin, vincristine (CAV) (Fukuoka *et al*, 1991; Roth *et al*, 1992). Carboplatin was shown to be active in the treatment of SCLC, and one randomised study comparing carboplatin and etoposide with PE showed no difference in survival, though the study was not powered for equivalence (Kosmidis *et al*, 1994).

When this clinical trial was designed in 1999, there were no published data from studies comparing ACE with a platinum/etoposide combination in patients with better-prognosis disease.

## Methods

The study design was a randomised phase III comparison of ACE with platinum/etoposide chemotherapy as first-line therapy in patients with better-prognosis SCLC.

### Eligibility criteria

Previously untreated patients with histologically or cytologically proven SCLC and a maximum of two adverse prognostic factors (extensive stage disease, PS ⩾2, raised LDH, serum sodium <130 *μ*mol l^−1^, Alk Phos >1.25 ULN) were eligible. Other eligibility criteria included age ⩾18 years, normal blood count, serum bilirubin <35 *μ*mol l^−1^ and creatinine clearance >50 ml min^−1^. In patients with impaired renal function, that is, creatinine clearance >30 ml min^−1^ but <50 ml min^−1^, and/or patients with significant cardiovascular disease, carboplatin could be substituted for cisplatin in the first or subsequent cycles.

A CT brain scan was not routinely performed, but patients with known brain metastases were not eligible.

The study had ethical and local approval and was covered by a DDX, later updated to a CTA after the introduction of EU Regulations. Patients gave their written informed consent. The Trial Management Committee consisted of the PI, the Co-Investigator, the lead research sister and the data manager. Adverse events were discussed by the TMC and those defined as serious and unexpected events were reported to the ethics committee.

After the publication in 2004 of two studies suggesting a survival benefit for platinum/etoposide in LD patients (Sundstrom *et al*, 2002; Thatcher *et al*, 2005), accrual of the final 12 patients required was limited to patients with extensive stage disease.

### Treatment and monitoring

Patients were randomised to receive six cycles of ACE (doxorubicin 50 mg/m^2^ i.v., cyclophosphamide 1 g/m^2^ i.v. and etoposide 120 mg/m^2^ i.v. on day 1, followed by etoposide 240 mg/m^2^ orally for 2 days) for 3 weeks or six cycles of PE (cisplatin 80 mg/m^2^ and etoposide 120 mg/m^2^ i.v. on day 1, followed by etoposide 240 mg/m^2^ orally for 2 days every 3 weeks). For patients where cisplatin was not suitable, carboplatin was substituted at an AUC of 6, calculated according to the Calvert formula (ie, carboplatin dose=target AUC of 6 (glomerular filtration rate+25 mg), where glomerular filtration rate was based on EDTA or measured creatinine clearance).

Chemotherapy was given if the total WBC was ⩾3000 *μ*/l, neutrophils ⩾1500 *μ*/l, platelets ⩾100 000 *μ*/l and creatinine clearance ⩾30 ml min^−1^, and there was no evidence of severe toxicity. If these conditions were not fulfilled, treatment was delayed and the blood count was repeated at intervals of not more than 1 week; treatment was given at full dose as soon as the above conditions were met. Dose reduction was not recommended. The use of GCSF as secondary prophylaxis was at the discretion of the investigator.

Thoracic radiotherapy was given to patients with limited stage disease achieving a complete or partial response to chemotherapy, beginning 3 weeks after the last cycle of chemotherapy (30 Gy in 10 daily fractions). Patients with ED SCLC received thoracic irradiation only if they had thoracic symptoms amenable to palliation with radiotherapy after completion of chemotherapy. Prophylactic cranial irradiation was considered for all LD patients achieving a complete response; suitable patients received 25 Gy in 10 daily fractions after completion of chemotherapy.

Tumour stage was assessed with CT scan of thorax and abdomen. Disease measurement was performed within 4 weeks before the start of treatment. During chemotherapy, patients were assessed on days 1 and 15 with physical examination, and weekly with blood count, biochemistry and WHO Performance Status. A chest X-ray (CXR) was carried out after every second cycle of treatment, but assessment of response was made according to the WHO criteria by CT scanning at the end of chemotherapy unless progressive disease was detected in the interim by CXR. Toxicities were graded according to the National Cancer Institute Common Toxicity Grading Criteria version December 1994 (revised).

### Statistical design

The study design was a randomised phase III comparison of ACE with platinum/etoposide as first-line therapy for patients with SCLC and a maximum of two adverse prognostic factors. The primary end point was 1-year survival. Secondary end points were 2-year survival, median survival, response rate and toxicity. Survival was calculated from the date of randomisation to the date of death from any cause. Time to progression was taken from the date of randomisation to the date of the progression. A 1-year survival of 40% had been reported for ACE in a recent MRC study (Thatcher *et al*, 2000). The North American experience suggested a 1-year survival rate of approximately 60% for a platinum-based combination, possibly in a more favourable patient group (Evans *et al*, 1987). Two hundred eighty patents were required to detect a survival difference of 20% (from 40 to 60%) at 1-year, with 90% power and a two-sided significance level of 5%. Patients were randomized on a 1 : 1 basis to one of two treatment arms. The allocation method was stochastic minimisation as implemented in a bespoke computer application at the randomisation centre. The only factor controlled for in the allocation was centre.

## Results

### Patient's characteristics

**Figure 1:**
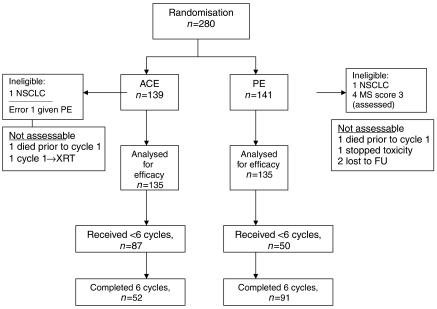
ACE/PE study flow diagram (CONSORT).

Between April 1999 and February 2005, 280 patients (ACE=139, PE=141) were randomised at two centres in the UK. The two arms were well balanced for age, stage, gender, PS and prognostic score (Table 1). All patients were included in the survival analysis on an intention to treat basis. Two patients were ineligible because of incorrect histological diagnosis (non-SCLC), one for each arm. A further seven patients were not assessable for response, three in the ACE arm (one patient received PE while was waiting for investigations, one died before cycle 1 and one needed radiotherapy following first cycle) and four patients in the PE arm (one died before cycle 1, one stopped the treatment after first cycle because of toxicity and two lost to follow-up after first cycle) (Figure 1).

### Treatment received

A total of 584 cycles of ACE and 696 of PE were administered (*P*=0.001) (Table 2). Fifty-two (37%) of the 139 patients randomised to ACE and 91 (65%) of patients randomised to platinum/etoposide completed all six cycles (*P*=0.01). The main reasons for early discontinuation were disease progression (ACE 19%, PE 12%) and toxicity (ACE 30%, PE 12%). Thirty-eight per cent of cycles of ACE were delayed or discontinued due to toxicity compared with 30% for PE (*P*=0.048). Six patients in the PE arm received carboplatin rather than cisplatin on cycle 1, and carboplatin was substituted for cisplatin during the treatment in a further four patients; one patient changed from PE to single-agent carboplatin.

In patients with limited stage disease, 58% patients in the ACE arm and 74% of patients in the PE arm received consolidation thoracic radiotherapy (*P*=0.04) and 23% of ACE patients and 33% of PE patients received PCI (*P*=0.24). A further 20 (14%) patients in the ACE arm and 12 (8.5%) in the PE arm received palliative radiotherapy. At disease progression, radiotherapy was given to 31 (22%) patients in the ACE arm and to 19 (13%) patients in the PE arm. Twenty-five (18%) and 21 (15%) of patients received second-line chemotherapy in the ACE arm and PE arm, respectively.

### Toxicity

Clinically significant toxicity was more common in patients receiving ACE (Table 3). Grades 3 and 4 anaemia occurred in 37 (27%) patients on the ACE arm and 25 (18%) patients on the PE arm (*P*=0.038). Grades 3 and 4 neutropenia occurred in 123 (90%) of patients receiving ACE and 78 (57%) of patients receiving PE (*P*<0.005). Neutropenia was associated with a high incidence of grades 3 and 4 infections, 73% of patients receiving ACE and 29% receiving PE arm (*P*<0.005). Eighty-two percent of patients in the ACE arm required i.v. antibiotics for one or more days during their treatment compared with 37% of PE patients. Hospitalisation for severe neutropenia and infections was less frequent with PE compared with ACE. The total number of days of hospitalisation for those treated with ACE was 1390 compared with 360 for PE (*P*<0.005). The median number of days of hospitalization was 9 (0–45) for ACE and 0 (0–18) for PE. Non-haematological toxicity was similar in both arms, but patients receiving PE experienced more grades 2 and 3 nausea.

### Response and survival

**Figure 2:**
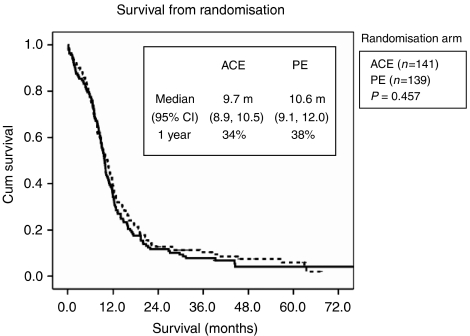
Survival (all patients).

The response rate was higher for PE patients, with a higher number of complete responders (Table 4). There was no significant difference in overall, median, 1-year or 2-year survival between the two treatment arms (Table 5 and Figure 2). For the LD group, the median survival was 10.9 months for ACE and 12.6 months for PE (*P*=0.58), with an actual 1-year survival rate of 44 and 54%, respectively (*P*=0.2). For the ED group, median survival was 8.3 months for ACE and 7.5 months for PE, with an actual 1-year survival rate of 17 and 15%, respectively. (*P*=0.9).

## Discussion

Although PE is now considered the treatment of choice for fitter patients with SCLC, this was not the case when the current trial was being designed. Several prospective studies had compared PE with anthracycline-based therapy, but most failed to demonstrate superiority of PE (Evans *et al*, 1987; Fukuoka *et al*, 1991; Roth *et al*, 1992). Subsequently, an overview of US National Cancer Institute sponsored trials for ED patients conducted between 1972 and 1990 demonstrated that cisplatin-based regimens were associated with an improved median survival (Chute *et al*, 1999) and a meta-analysis of 19 trials published between 1981 and 1999 showed a small survival benefit for patients receiving cisplatin-based chemotherapy (Pujol *et al*, 2001). Etoposide-containing regimens, with or without cisplatin, were also shown to be associated with a significant survival benefit (Mascaux *et al*, 2000).

After this study had begun accrual, a trial from Norway comparing PE with cyclophosphamide, epirubicin, etoposide (CEV) reported a survival benefit for PE (Sundstrom *et al*, 2002). The median survival was 7.8 months for CEV and 10.2 months for PE, with a 2-year survival of 6 and 14%, respectively (*P*=0.0004). The advantage was confined to patients with LD, with a median survival of 14.5 months for PE and 9.7 months for CEV (*P*=0.001), and a 2-year survival of 25 and 10% (*P*=0.0001), respectively. There was a trend in the same direction for patients with ED, but this was not statistically significant. Although the proportions of patients with extensive stage disease and with PS2 were similar in both studies, the choice and doses of drugs are not directly comparable. The comparator arm in the Norwegian study used a lower dose of anthracycline (epirubicin 50 mg/m^2^ *vs* doxorubicin 50 mg/m^2^) and used vincristine rather than etoposide. The median survival for PE was similar in both studies (10.6 *vs* 10.2 months). The different survival seen for the two-comparator arms (7.8 months for CEV *vs* 9.6 months for ACE) may explain in part the statistically significant survival advantage seen for PE in the Sundstrom study. The survival for ACE in this study is marginally lower than that reported for two studies from the UK Medical Research Council, but these studies had fewer ED patients (Thatcher *et al*, 2000; Thatcher *et al*, 2005).

The median and 2-year survivals for PE in LD patients in our study were lower than those reported by Sundstrom (12.6 *vs* 14.5 months, 21 *vs* 25%, respectively). This may in part reflect lower use of radiotherapy, which has an established role in consolidating the response of the primary tumour to chemotherapy and in reducing the risk of brain metastases as a site of recurrence. (Pignon *et al*, 1992; Warde and Payne, 1992). Cisplatin plays a central role in concurrent treatment, because of its radiosensitising effect, and there is increasing evidence of a survival benefit for patients receiving early, concurrent, cisplatin-based chemoradiotherapy with 5-year survival ranging between 20 and 25% (Fried *et al*, 2004; Pijls-Johannesma *et al*, 2005). In this study, thoracic radiotherapy was given after the completion of chemotherapy. The rate or TRT was lower for patients receiving ACE (58%) than those receiving PE (74%). The reason for this is unclear, but is likely to reflect the worse toxicity seen with ACE, and is similar to the 51–54% reported in comparable studies (Thatcher *et al*, 2000; Ardizzoni *et al*, 2002). Higher TRT rates have also been reported for other platinum combinations (Lorigan *et al*, 2005; Thatcher *et al*, 2005). The TRT rate in the Sundstrom study was higher in both arms (83 and 88%). Prophylactic cranial irradiation has been shown to be associated with a survival advantage in LD and ED patients responding to chemotherapy (Auperin *et al*, 1999) (Slotman *et al*, 2007). In this study, 33% of LD patients receiving PE and 23% of those receiving ACE went on to have PCI. The use of PCI was comparable with the Sundstrom study, but lower than that reported in studies using VICE (35–53%) (Lorigan *et al*, 2005; Thatcher *et al*, 2005).

We observed that haematological toxicity and the risk of infection were significantly higher for ACE than for PE. This difference was clinically relevant, with a higher rate of grades 3 and 4 infection, higher number of days in hospital and higher rate of treatment discontinuation for ACE. The incidence of grades 3 and 4 infection in this study was 73% for ACE, and 29% for PE. Intravenous antibiotics were used in 82% of patients receiving ACE and 37% of patients receiving PE. The infection rates seen in both arms of this study were substantially higher than the 14–15% reported for ACE and 16% for VICE – toxicity data were not reported for the Sundstrom study. (Thatcher *et al*, 2005; Thatcher *et al*, 2000) However, the toxic death rate (1%) is lower than the 4–10% reported in other comparable studies (Thatcher *et al*, 2000; Pujol *et al*, 2001; Ardizzoni *et al*, 2002). The likely explanation for the high-recorded infection rate but low toxic death rate in both arms was that patients were seen weekly and clinical teams had a low threshold for responding to neutropenia. Other factors (low use of GCSF, lack of use of prophylactic antibiotics, use of oral etoposide, higher proportion of ED patients) may also have contributed to the proportionally higher infection rate seen in both arms.

The study was initially designed to allow inclusion of patients not fit for cisplatin chemotherapy, allowing these to be treated with carboplatin. We were surprised by the low number of patients (six) who commenced treatment with carboplatin. A further four patients changed to carboplatin during treatment. We have included these patients with cisplatin for both efficacy and toxicity analysis.

Further advances in the treatment of patients with SCLC are most likely to come with optimum use of concurrent chemotherapy and radiotherapy, and the identification of new drugs and targets. Until then, the combination of cisplatin and etoposide is standard therapy for patients with SCLC and good PS, and further studies of traditional anthracycline-based regimens are not warranted.

## Figures and Tables

**Table 1:** Patient and tumour characteristics

	**ACE**	**PE**	**Total**	***P*-value**
No. of patients	139	141	280	
Male/female	67/72	75/66	142/138	0.47
Median age, years (range)	66 (38–81)	65 (39–89)		0.95
				
*Stage*				
Limited	84 (60)	81 (57)	165	0.65
Metastatic	54 (39)	60 (43)	112	
				
Limited stage, good PS, normal LDH	21%	29%		
				
*Prognostic score (MS)*				
0	31 (22)	41 (29)	72	
1	63 (45)	56 (40)	119	
2	45 (33)	40 (28)	85	
3	0 (0)	4 (3)	4	0.1
				
*Performance status (WHO)*				
0	19 (14)	20 (14)	39	
1	82 (59)	83 (59)	165	
2	36 (26)	37 (26)	73	
3	1 (1)	1 (1)	2	

**Table 2:** Number of cycles treatment administered

	**ACE**	**PE**	
***N***	**137 (%)**	**140 (%)**	***P*-value**
*No. of cycles*			
0	2 (2)	1 (1)	
1	18 (13)	11 (8)	
2	11 (8)	7 (5)	
3	14 (10)	11 (8)	
4	20 (14)	8 (6)	
5	22 (16)	12 (9)	
6	52 (37)	91 (65)	0.001
Total	584	696	

**Table 3:** Toxicity

	**ACE (*N*=137), no. of patients (%)**		**PE (*N*=140), no. of patients (%)**	
**Toxicity**	**Grades 1 and 2**	**Grades 3 and 4**	**Grades 1 and 2**	**Grades 3 and 4**
*Haematologic adverse events*				
Anaemia	88 (64)	38 (28)	96 (69)	27 (19)
Neutropenia	3 (2)	125 (91)	47 (34)	82 (59)
Thrombocytopenia	36 (26)	77 (56)	39 (28)	69 (49)
				
*Non-haematologic adverse events*				
Infection	13 (9)	100 (73)	24 (18)	40 (39)
Nausea	65 (47)	06 (4)	75 (55)	13 (9)
Vomiting	41 (30)	3 (2)	52 (38)	8 (6)
Constipation	37 (27)	2 (1)	55 (41)	4 (3)
Oral mucositis	63 (46)	9 (7)	50 (37)	2 (1)
Lethargy	80 (59)	13 (9)	96 (70)	14 (10)
Anorexia	68 (50)	8 (6)	76 (56)	5 (4)
Alopecia (grades 1–3)	42 (31)	52 (38)	65 (48)	72 (51)
Hoarse voice	35 (26)	2 (1)	27 (20)	6 (4)
Neuropathy	16 (12)	0	29 (21)	1 (1)
Cough	84 (61)	1 (1)	84 (62)	4 (3)
				
		***N*=137 (%)**	***N*=136 (%)**	***P*-value**
*Intravenous/oral antibiotics during all cycles*				
No. of patients	None	17 (12)	67 (49)	
	Oral only	3 (2)	17 (13)	<0.005
	Intravenous only	52 (38)	21 (16)	
	Intravenous+oral	60 (44)	29 (21)	
	NK	5 (4)	2 (1)	
No. of cycles	Affected/	243/578	76/685	<0.005
	possible (%)	(42)	(11)	
No. of days	Affected/	1390/12138	360/14385	<0.005
	possible (%)	(11)	(3)	

NK=not known.

**Table 4:** Response to treatment (*N*=280)

**Response to treatment**	**ACE (%)**	**PE (%)**
Complete response	25 (18)	36 (26)
Partial response	72 (53)	70 (50)
stable disease	6 (4)	13 (9)
Progressive disease	27 (20)	17 (12)
Toxic death	2 (1)	1 (1)
Not assessable	5 (4)^a^	3 (2)^b^

a One patient died before cycle 1; one had PE; one had XRT following first cycle; one died after cycle 1 due to toxicity not assessed; one stopped after cycle 1 due to toxicity.

b One patient stopped after cycle 1 due to toxicity not assessed; two lost to FU.

**Table 5:** Survival

	**ACE**	**PE**
*All Patients*		
Median survival (months)	9.7	10.6
1-year survival (%)	34	38
2-year survival (%)	12	12
		
*LD*		
Median survival (months)	10.9	12.6
1-year survival (%)	44	54
2-year survival (%)	19	16
		
*ED*		
Median survival (months)	8.3	7.5
1-year survival (%)	17	15
2-year survival (%)	0	3
